# Conventional Transarterial Chemoembolization Versus Drug-Eluting Beads in Patients with Hepatocellular Carcinoma: A Systematic Review and Meta-Analysis

**DOI:** 10.3390/cancers13246172

**Published:** 2021-12-07

**Authors:** Khalid I. Bzeizi, Mohammad Arabi, Negar Jamshidi, Ali Albenmousa, Faisal M. Sanai, Waleed Al-Hamoudi, Saad Alghamdi, Dieter Broering, Saleh A. Alqahtani

**Affiliations:** 1 King Faisal Specialist Hospital & Research Center, Riyadh P.O. Box 3354, Saudi Arabia; albenmousa@kfshrc.edu.sa (A.A.); walhamoudi@kfshrc.edu.sa (W.A.-H.); mdisaad@kfshrc.edu.sa (S.A.); dbroering@kfshrc.edu.sa (D.B.); salqaht1@jhmi.edu (S.A.A.); 2 Department of Oncology, Ministry of National Guard Health Affairs, King Abdulaziz Medical City, Riyadh P.O. Box 22490, Saudi Arabia; marabi2004@hotmail.com; 3 School of Science, RMIT University, Bundoora, VIC 3083, Australia; z5340023@unsw.ad.edu.au; 4 Gastroenterology Unit, Department of Medicine, King Abdulaziz Medical City, Jeddah P.O. Box 9515, Saudi Arabia; sanaifa@ngha.med.sa; 5 Division of Gastroenterology & Hepatology, Johns Hopkins University, Baltimore, MD 21218, USA

**Keywords:** hepatocellular carcinoma, drug-eluting beads, transarterial chemoembolization

## Abstract

**Simple Summary:**

Hepatocellular carcinoma (HCC) is the most common type of liver cancer and accounts for approximately 6% of all human cancers. In this study, we performed a systematic review and pooled analysis of the conventional transcatheter arterial chemoembolization (C-TACE) compared to drug-eluting beads TACE (DEB-TACE) as two treatment options for patients with unresectable HCC. Treatment with DEB-TACE appears to be non-inferior compared to conventional C-TACE and associated with a better objective response and disease control with fewer severe complications and all-cause mortality. In light of these findings, research efforts should attempt to further characterize the efficacy and safety profile of DEB-TACE as a potential component of unresectable HCC management.

**Abstract:**

Hepatocellular carcinoma (HCC) occurs in nearly three-quarters of all primary liver cancers, with the majority not amenable to curative therapies. We therefore aimed to re-evaluate the safety, efficacy, and survival benefits of treating patients with drug-eluting beads transcatheter arterial chemoembolization (DEB-TACE) compared to the conventional transcatheter arterial chemoembolization (C-TACE). Several databases were searched with a strict eligibility criterion for studies reporting on adult patients with unresectable or recurrent HCC. The pooled analysis included 34 studies involving 4841 HCC patients with a median follow-up of 1.5 to 18 months. There were no significant differences between DEB-TACE and C-TACE with regard to complete response, partial response and disease stability. However, disease control (OR: 1.42 (95% CI (1.03,1.96) and objective response (OR: 1.33 (95% CI (0.99, 1.79) were significantly more effective for DEB-TACE treatment with fewer severe complications and all-cause mortality. The pooled-analysis did not find superiority of DEB-TACE in complete or partial response, disease stability, controlling disease progression, and 30 day or end-mortality. However, results showed that DEB-TACE is associated with a better objective response, disease control, and lower all-cause mortality with severe complications compared to C-TACE treatment. Given that the safety outcomes are based on limited studies with a potential for bias, there was no clear improvement of DEB-TACE over C-TACE treatment.

## 1. Introduction

Liver cancer ranks as the sixth most common cancer and fourth major cause of cancer-related deaths globally [1]. An estimated 841,000 newly diagnosed liver cancers were reported in 2018, with numbers projected to increase by 60% to 1.36 million cases by 2040, and annual deaths (782,000) are set to increase by over 64% to 1.28 million by 2040 [2]. Hepatocellular carcinoma (HCC) is a complex condition accounting for more than three-quarters of all primary liver cancers and approximately 6% of all human cancers [1,3,4]. Given that early detection of HCC is uncommon and the majority of patients are diagnosed at the intermediate or advanced stage of the disease, typically with dismal prognosis, HCC has a significantly high mortality rate [5,6].

Among the supportive therapies, conventional transcatheter arterial chemoembolization (C-TACE) is recommended as the first-line palliative treatment option for patients with unresectable HCC [7]. Despite it being an effective approach in treating intermediate HCC, there are some major drawbacks to C-TACE therapy [8,9,10]; the C-TACE typically uses iodized oil-based emulsion (lipiodol oil) with chemotherapeutic drugs, which enters the systemic circulation and can result in a higher incidence of systemic adverse effects [11]. Furthermore, in some patients, the lipiodol oil emulsion in C-TACE has been associated with severe pain [7]. In order for the systemic side effects of chemotherapy to be reduced, TACE with drug-eluting beads (DEB-TACE) has been recently developed, which promotes the selective and controlled delivery of cytotoxic drugs [12]. The DEB-TACE contains microspheres that can be loaded and then release high concentrations of a variety of drugs in tumor tissues in a controlled and sustained manner without concomitant elevation in systemic concentrations [8,13].

While a number of studies have evaluated the safety and efficacy of DEB-TACE for treating unresectable HCC [14,15,16,17,18], relatively fewer prospective and retrospective studies have compared C-TACE to DEB-TACE [19,20,21]. More recent clinical studies [22,23], as well as several meta-analyses [24,25,26,27,28,29,30,31] comparing these two treatment modalities, provide inconsistent results, with some reporting no significant differences in tumor response rate between the two in managing patients with unresectable HCC [22,23,25,27], while others found a greater overall survival rate, as well as tumor response in DEB-TACE-treated patients with HCC compared to those with C-TACE treatment [24,26].

Therefore, the overarching aim of this meta-analysis was to re-evaluate the safety, efficacy, and survival benefit of DEB-TACE compared to C-TACE treatment for managing patients with unresectable HCC. Given that recent meta-analyses have focused only on randomized trials [30], have posed no restriction on stage of disease [31], or have reported only some of the outcomes [31], we aimed to include both observational and controlled trials as well as all reported outcomes with a focus on unresectable or recurrent HCC.

## 2. Materials and Methods

This systematic review and meta-analysis were performed according to the Preferred Reporting Items for Systematic reviews and Meta-Analysis (PRISMA) guidelines [32]. The protocol has been registered in Research Registry (https://www.researchregistry.com: ID number reviewregistery1236) on 16 October 2021.

### 2.1. Eligibility Criteria for Inclusion of Studies

Studies were deemed eligible and included in this analysis if they included adult patients with unresectable de novo or recurrent HCC without extrahepatic spread and who did not receive adjuvant treatment with systemic chemotherapy or concurrent locoregional radiofrequency ablation or ethanol injection. Moreover, studies that included patients who were previously treated with either C-TACE or other locoregional treatment prior to the inclusion in the study were eligible for this review.

The intervention and control evaluated in this review were DEB-TACE and C-TACE, respectively. Studies that reported any of the following efficacy and safety outcomes were included in this analysis: complete response, partial response, objective response, disease stability, disease control, and disease progression. Moreover, studies reporting safety outcomes of serious adverse events, systemic side effects, 30-day mortality, and end mortality were included.

Tumor response assessments in the included studies were reported according to the European Association for the Study of the Liver (EASL) system and modified Response Evaluation Criteria in Solid Tumors (mRECIST) systems (see Table S1). The former system is a bidimensional approach that assesses the largest diameters of the enhancing viable tumor on the arterial phase of triphasic liver computed tomography, while the latter is a unidimensional approach that calculates the largest axial diameter of the viable enhancing tumor.

Studies were eligible for inclusion if they compared C-TACE with DEB-TACE technique for management of patients with unresectable HCC. The initial aim was to include only randomized controlled trials (RCTs) for analysis; however, owing to the limited number of RCTs, this scope was broadened. Therefore, we decided to include cohort and case–control studies in an effort to sufficiently address the review objectives. Other observational studies, such as case reports, case series, and reviews, were excluded.

### 2.2. Search Methods

The databases searched included Cochrane Library, Medline, Embase, and PubMed from inception to November 2021. In addition, manual searches of reference lists of review articles and relevant studies were performed. The full-texts of any references identified as potentially eligible were also retrieved. In order to identify unpublished and ongoing studies, we also searched the registry of ClinicalTrials.gov. Search terms included: hepatocellular carcinoma, unresectable, liver tumor, transarterial chemoembolization, TACE, C-TACE, lipiodol, cisplatin, doxorubicin, epirubicin, DC beads, biocompatibles, and DEB-TACE (for detailed search strategy, refer to Table S2).

### 2.3. Selection of Studies

Two authors independently screened titles and abstracts for potential studies (K.B. and A.A.). Any disagreement between the authors’ opinions were resolved by discussion or, if necessary, through consultation with a third author (M.A.). The full-texts of studies that fulfilled the inclusion criteria were retrieved from initial screening. Two independent authors (K.B. and A.A.) assessed the eligibility of each study and reached a resolution, as described above.

### 2.4. Data Extraction

The following data were extracted on a predefined data extraction sheet created in Microsoft Excel: participant characteristics, study interventions, study outcome measures, and information on the design and methodology of the trials. Data extraction from the retrieved final evaluation were performed by one author (A.A.) and reviewed by a second author (M.A.).

### 2.5. Statistical Analysis

For binary outcome variables, the outcome measure was calculated as odds ratio (OR) together with the 95% confidence interval (CI). Pairwise meta-analysis was performed between two similar interventions, which were used in more than two trials, by using a random effects model. Statistical analysis for all the variables were conducted with R software (R version 3.5.2) by using meta-package, and *p* < 0.05 was considered as statistically significant.

### 2.6. Testing for Heterogeneity

A quantitative estimate of statistical heterogeneity between studies was assessed using the tau square (τ^2^) statistics. The I^2^ statistic was used to quantify the level of heterogeneity that was interpreted as low, medium, and high when I^2^ was 25%, 50%, and 75%, respectively. To accommodate between study heterogeneity, we used the Dersimonian and Laird random-effect model for variables [33].

### 2.7. Assessment of Publication Bias

Publication bias was assessed through funnel plots. In terms of the shape of the graph, a symmetrical graph indicated the absence of publication bias, whereas an asymmetrical graph indicated the presence of publication bias. Egger’s weighted regression was used to confirm publication bias [34].

## 3. Results

### 3.1. Identification of Eligible Studies

**Figure 1:**
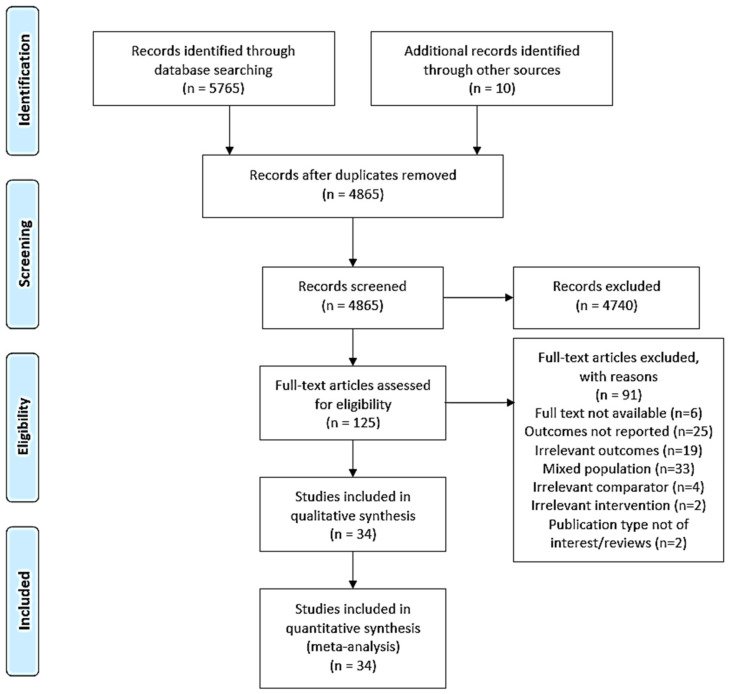
PRISMA flow diagram of the process for study selection.

Following a search of several electronic databases, a total of 5775 potentially relevant studies were identified for initial screening. After removing the duplicates (*n* = 910), the remaining 4865 studies were screened on the basis of title and abstract, which resulted in the exclusion of irrelevant studies (*n* = 4740). From screening the remaining full-text studies (*n* = 125), a total of 34 studies were retrieved and included in this meta-analysis (Figure 1).

### 3.2. Study Characteristics

The included studies were 5 RCTs, 20 cohort studies, and 9 case–control studies, which were mainly conducted between 2010 and 2021. A total of 4841 patients with age range between 25 and 91 years were included in the meta-analysis. Most of the participants were males with a median follow-up of 1.5 to 18 months. Patients who received DC beads (*n* = 2283) were comparable to those treated with C-TACE (*n* = 2558). Full details of the included studies are explained in Table 1.

### 3.3. Efficacy Outcomes

The summary of pooled analyses for efficacy and safety outcomes are presented in Table 2, together with publication bias summaries where less than 10 studies were available for the pooled analysis (Table 2).

#### 3.3.1. Complete Response

**Figure 2:**
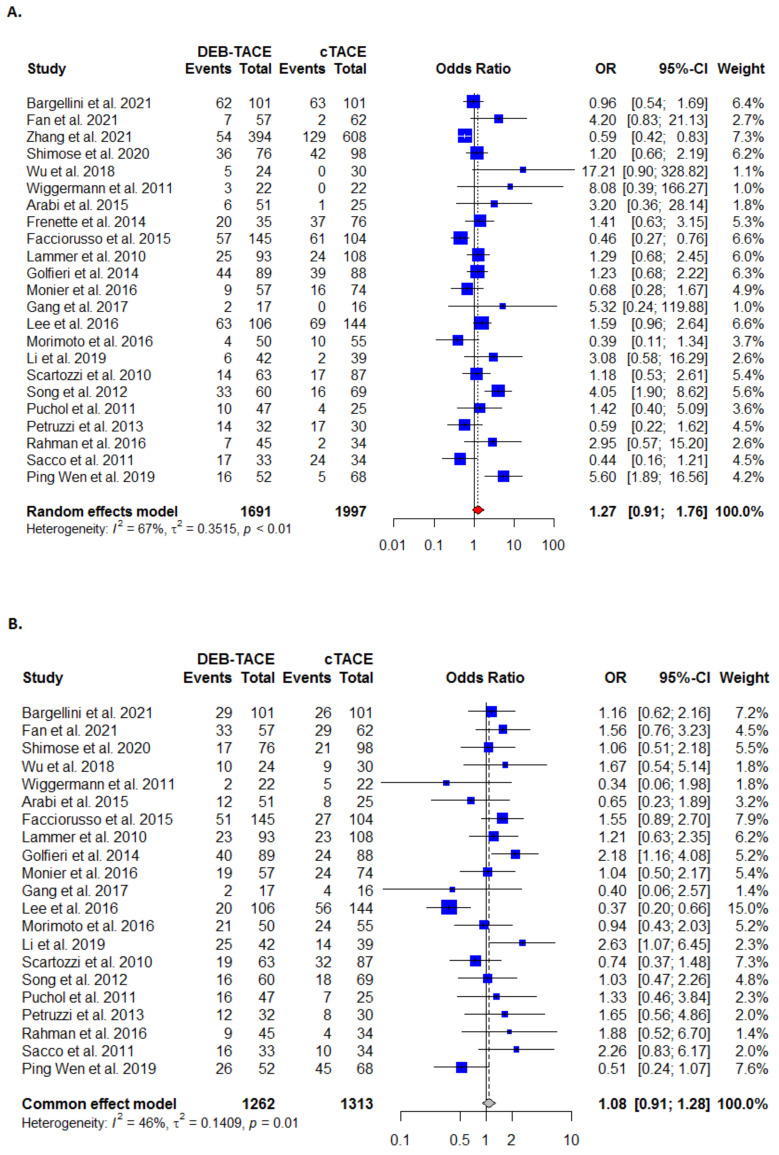

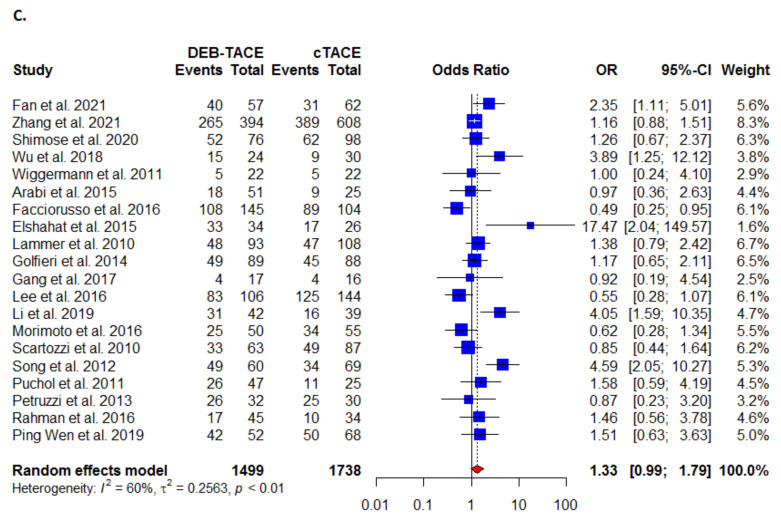
Forest plots for (**A**) complete response analysis; (**B**) partial response analysis; (**C**) objective response analysis between DEB-TACE (experimental) and c-TACE (control) treatment in patients with hepatocellular carcinoma.

Complete response was reported in 23 studies. Although more patients in DEB-TACE achieved complete response than in the C-TACE control arm (Figure 2A, Table 2), the treatment difference was not statistically significant (OR: 1.27; 95% CI (0.91, 1.75). There was significant heterogeneity noted for this outcome (I^2^ = 67%; τ^2^ = 0.3515, *p* < 0.01).

#### 3.3.2. Partial Response

The partial response outcome was reported in 21 studies. The DEB-TACE was found to be more effective than the C-TACE (Figure 2B, Table 2), but the treatment difference was not statistically significant (OR: 1.08 (95% CI (0.91, 1.28). There was significant heterogeneity present (I^2^ = 46%; τ^2^ = 0.1409, *p* < 0.05).

#### 3.3.3. Objective Response

The objective response outcome was reported in 20 studies. The DEB-TACE was reported significantly more effective than C-TACE (OR: 1.33 (95% CI [0.99, 1.79]) (Figure 2C, Table 2). Heterogeneity was moderate and statistically significant (I^2^ = 60%; τ^2^ = 0.2563, *p* < 0.01).

#### 3.3.4. Disease Stability

**Figure 3:**
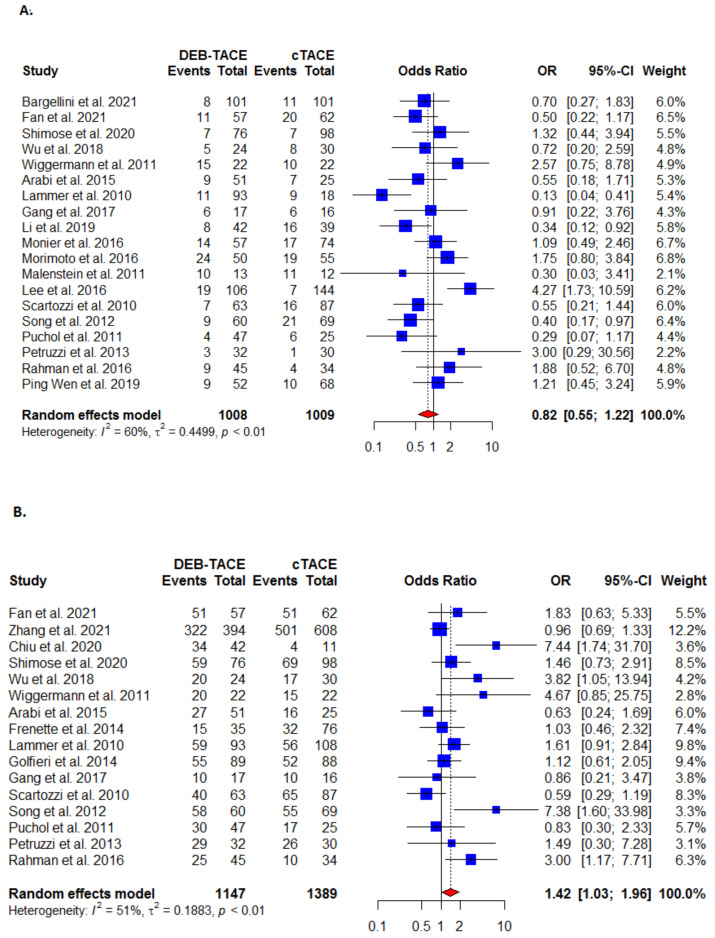

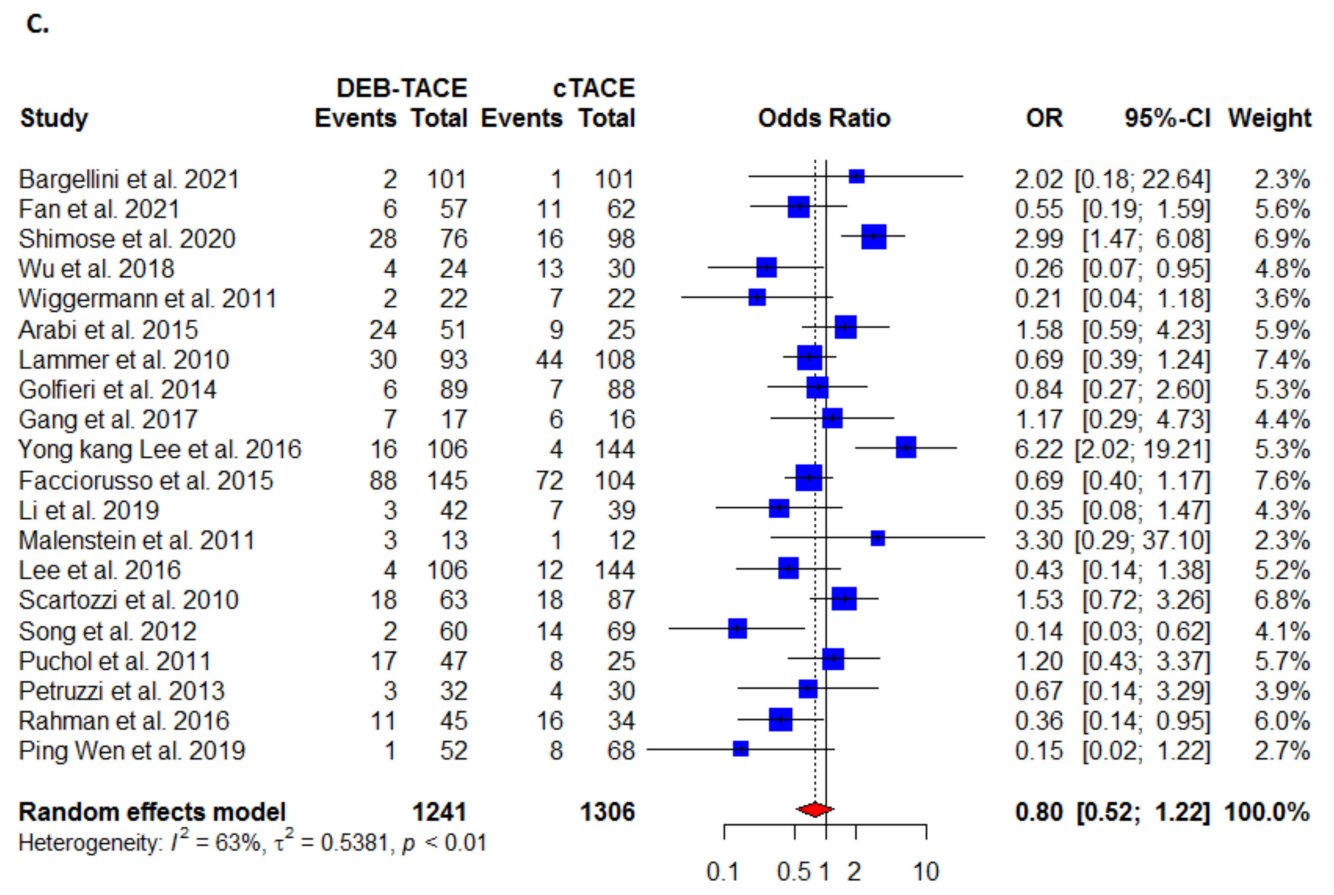
Forest plots for (**A**) disease stability; (**B**) disease control; (**C**) disease progression analysis between DEB-TACE (experimental) and C-TACE (control) treatment in patients with hepatocellular carcinoma.

The disease stability outcome was reported in 19 studies, with DEB-TACE more effective than the C-TACE (Figure 3A, Table 2), but the treatment difference was not statistically significant (OR: 0.82 (95% CI [0.55, 1.22]). There was statistically significant heterogeneity present (I^2^ = 60%; τ^2^ = 0.4499, *p* < 0.01).

#### 3.3.5. Disease Control

The disease control outcome was reported in 16 studies where the DEB-TACE was found to be significantly more effective than the C-TACE (OR: 1.42 (95% CI [1.03, 1.96]) (Figure 3B, Table 2). There was statistically significant heterogeneity detected (I^2^ = 51%; τ^2^ = 0.1883, *p* < 0.01).

#### 3.3.6. Disease Progression

The disease progression outcome was reported in 20 studies where the DEB-TACE was non-significantly less effective than the C-TACE (OR: 0.80; 95% CI [0.52, 1.22]) (Figure 3C, Table 2). There was statistically significant heterogeneity observed (I^2^ = 63%; τ^2^ = 0.5381, *p* < 0.01).

### 3.4. Safety Outcomes

#### 3.4.1. Systemic Side Effects

**Figure 4:**
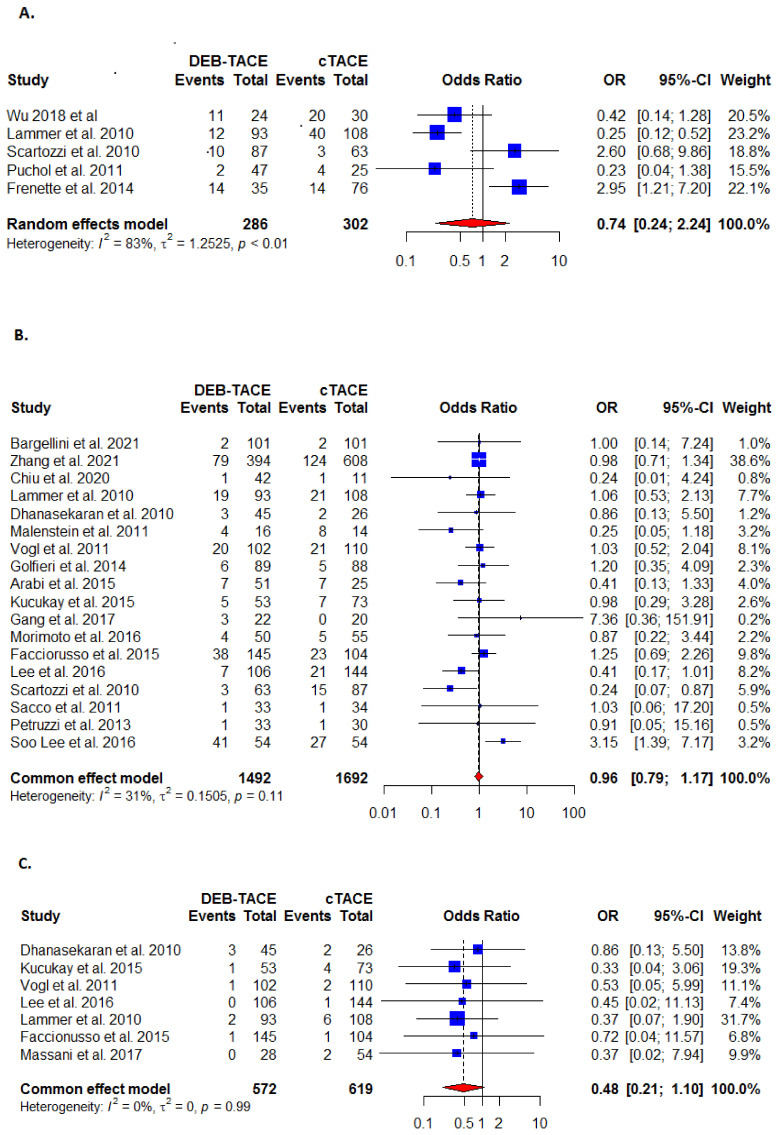

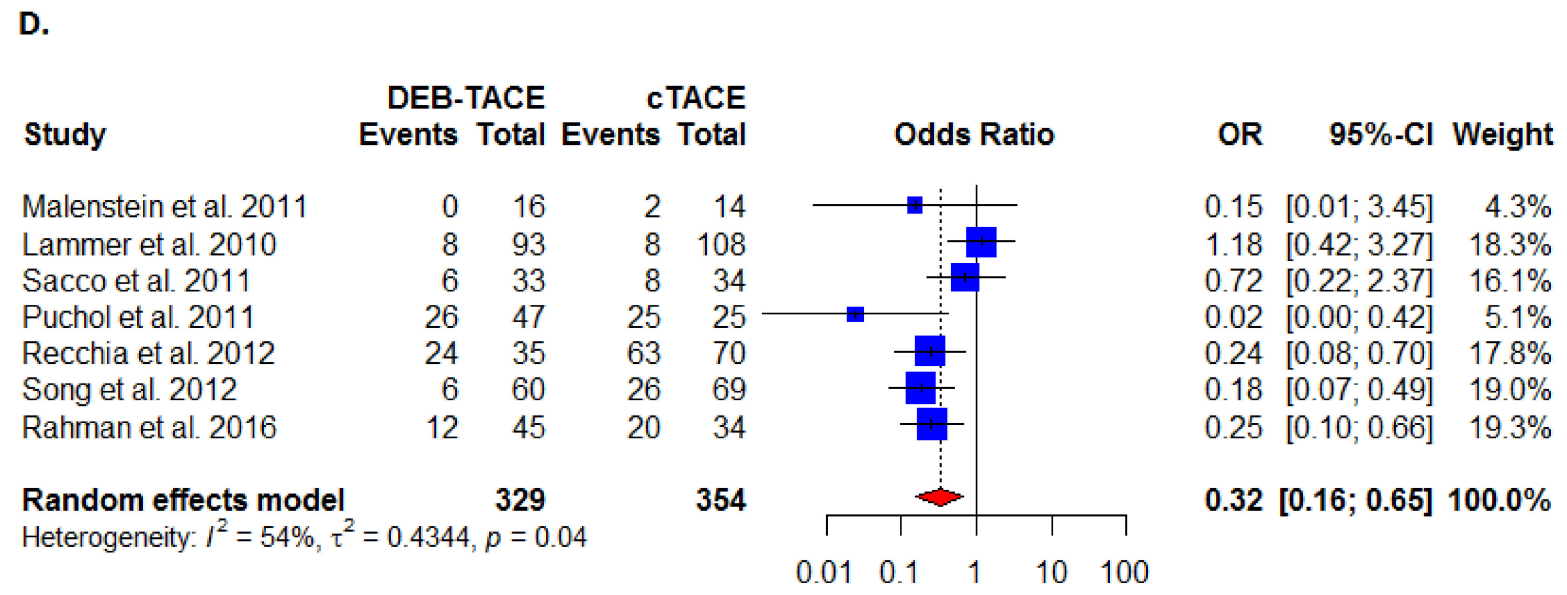
Forest plots for safety outcomes between DEB-TACE (experimental) and C-TACE (control) treatment in patients with hepatocellular carcinoma (**A**) systemic effects; (**B**) serious adverse events; (**C**) 30-day mortality; (**D**) end mortality.

The systemic side effects outcome was reported in five studies and found to be lower in the DEB-TACE- than in the C-TACE-treated patients (Figure 4A, Table 2), but the difference was not statistically significant (OR: 0.74 (95% CI [0.24, 2.24]). There was statistically significant heterogeneity present (I^2^ = 83%; τ^2^ = 1.2525, *p* < 0.01).

#### 3.4.2. Serious Adverse Events

The outcome of serious adverse events was reported in 18 studies and found to be in the DEB-TACE- than the C-TACE-treated patients (Figure 4B, Table 2), but the difference was not statistically significant (OR: 0.96 (95% CI [0.79, 1.17]). No significant heterogeneity was reported (I^2^ = 31%; τ^2^ = 0.1505, *p* = 0.11).

#### 3.4.3. Thirty-Day Mortality

The 30-day mortality outcome was reported in seven studies where the DEB-TACE-treated patients were significantly safer than in C-TACE-treated patients (OR: 0.48; 95% CI [0.21, 1.10]) (Figure 4C, Table 2). Heterogeneity was moderate (I^2^ = 0%; τ^2^ = 0, *p* = 0.99).

#### 3.4.4. End Mortality

The end mortality outcome was also reported in seven studies, with the DEB-TACE treatment non-significantly safer than in C-TACE treated patients (OR: 0.32 (95% CI [0.16, 0.65]) (Figure 4D, Table 2). There was statistically significant heterogeneity was identified (I^2^ = 54%; τ^2^ = 0.4344, *p* < 0.05).

### 3.5. Publication Bias

The Egger’s test and funnel plot showed significant publication bias for the complete response (*p*-value < 0.05) and disease control (*p*-value < 0.05). However, there was no significant publication bias for partial response (*p*-value = 0.97), objective response (*p*-value = 0.23), disease stability (*p*-value = 0.75), disease progression (*p*-value = 0.42), and serious adverse events outcomes (*p*-value = 0.42). The “trim and fill” method was used for adjusting publication bias but did not show potentially missing studies for this meta-analysis.

## 4. Discussion

This systematic review with meta-analysis assessed the potential benefits of DEB-TACE compared to C-TACE in the management of patients with unresectable HCC and explored the differences in patient cohorts that may have impacted the outcomes of individual studies. Our analysis suggests that DEB-TACE is associated with a better objective response and disease control, whereas the pooled analysis showed no significant benefit of DEB-TACE in complete or partial response, disease stability, controlling disease progression, and 30 day or end mortality outcomes. Moreover, our findings revealed that DEB-TACE resulted in fewer severe complications and it appeared to be associated with less 30 day and all-cause mortality in the examined studies.

A number of studies report that the incidence of serious post-treatment liver toxicity following DEB-TACE is lower, with recorded increases in the aspartate and alanine aminotransferase levels compared to the levels in the C-TACE-treated patients [15,19,21]. Our meta-analysis findings are consistent with some of the recent analyses, which have shown that DEB-TACE has a comparable safety profile to C-TACE [30] and provides a significantly better objective tumor response than in C-TACE-treated patients [27,28], with higher 1 and 2 year survival rates in DEB-TACE-treated patients [24,28]. Although pooled and subgroup analysis of RCTs vs. non-RCTs have shown no statistically significant difference between the two treatments, one RCT [17] and one observational study [18] published during the same search period were not included, which may have resulted in underpowered analysis. However, a recent meta-analysis of six RCTs did not find significant differences between these two treatments in overall survival or major complications and concluded that the safety and efficacy profile of DEB-TACE was comparable to that of the C-TACE profile [30]. In another recent meta-analysis, although the study also found comparable overall survival and adverse events between C-TACE and TACE with CalliSpheres^®^ microspheres (CSM), a polyvinyl alcohol hydrogel microsphere, the pooled analysis revealed that CSM-TACE showed significantly superior efficacy outcomes compared to that in C-TAC-treated patients [31]. These differences are likely due to heterogeneity between study populations included in the pooled analyses and differences in methodological designs.

Regarding systemic toxicity, our review showed lower incidence of systemic toxicity and improved tolerance with DEB-TACE, similar to the findings of the Precision V RCT for intermediate, as well as more advanced patients with HCC, which was based on multiple stratification factors [21]. The lower risk for systemic toxicity allows for the use of higher doxorubicin dosage, which does not appear to be associated with a higher incidence of major chemotherapy-related adverse events [35].

Dhanasekaran et al. found that patients with portal vein thrombosis and Child–Pugh A and B liver disease did not develop liver failure as a result of either treatment, suggesting that DEB-TACE could be safely administered in this subset of patients [20]. However, the study findings from subgroup analysis revealed no significant differences in long-term survival between patients with patent and thrombosed portal veins following DEB-TACE treatment [20]. Our analysis is inherently limited by the substantial heterogeneity of evaluated studies, specifically related to the characteristics of patients with HCC, variability of treatment protocols, and response assessment methodologies. Since the study design was significantly heterogeneous across the included studies, we did not assess the quality of the included studies. The Precision V study included patients with more advanced disease in both study arms, specifically patients with higher BCLC classification, Child–Pugh score, and bilobar disease [21]. A small percentage of the patients in the Precision V study received curative therapy prior to the randomization to one of the treatment arms. The authors concluded that DEB-TACE is safe and effective in the treatment of HCC and offers a benefit to patients with more advanced disease. A later trial by Sacco et al. included a greater proportion of early stage tumor (approximately 65% BCLC A) and significantly smaller mean tumor size for both treatment arms [16]. This RCT also excluded all patients with previous treatments and included only patients with untreated HCC lesions. These factors may explain the higher rates of complete and partial response with C-TACE in this highly selected cohort of patients and suggest that DEB-TACE could be of better value in advanced stages of the disease. Dhanasekaran et al., who included patients with Child–Pugh C class and portal vein thrombosis, further showed that DEB-TACE improves survival in patients with Child–Pugh A and B classes without survival benefit in Child–Pugh C class or advanced tumor stages [20]. While patients who developed liver failure after DEB-TACE belonged to Child–Pugh C, liver failure occurred in patients treated with C-TACE in classes A and B.

Treatment protocols across the DEB-TACE studies were more consistent, particularly in terms of the embolization material, beads size, and chemotherapy doses. However, C-TACE protocols varied widely among reported studies. This difference may have impacted standardization of outcomes and appropriate comparisons across the included studies [36].

The current study has considerable strengths over more recently published meta-analyses [30,31]. In our review, we have pooled all available evidence published to date including RCTs and observational studies, whereas Wang et al. (2020) included only evidence from randomized trials. While we restricted our eligibility criteria to include studies that only reported unresectable or recurrent HCC, the study design by Liang et al. (2021) did not pose any restrictions on stage of the disease. Furthermore, these authors did not report other important outcomes for disease progression, disease stability, and mortality rates, which we have reported in our meta-analysis inclusively. Moreover, the most recent meta-analysis restricted publications to English and Chinese languages until March 2020 [31], whereas we did not apply any language restrictions and extended our search to November 2021.

Finally, several limitations may contribute to our findings of treatment outcomes and safety. The hyperdense lipiodol used in C-TACE may falsely mask any residual or recurrent enhancing tumor on the follow-up computed tomography scan, whereas lesion detection can be easily achieved in patients who received DEB-TACE due to the absence of adjacent hyperdensity [37]. This may spuriously improve the tumor response in C-TACE cases, leading to under-treatment, and, in turn, may improve patient outcomes in DEB-TACE cases. The findings of safety profile need to be interpreted with caution as relatively small number of studies reported safety outcomes, which might present as a potential source of bias. The short median duration of follow-ups (range: 1.5–12 months) might have decreased the statistical power and more studies of longer follow-up durations are warranted to validate these findings.

## 5. Conclusions

Although the current review of treatment efficacy is marginally in favor of DEB-TACE, while not strongly supporting or refuting its superiority over C-TACE in treatment of unresectable HCC, the findings within discussed limitations suggest that DEB-TACE is associated with fewer side effects, as well as a lower incidence of liver and systemic toxicity. Given that safety findings are based on very limited data with a potential for bias, no clear improvement of efficacy of DEB-TACE over C-TACE is present. These findings warrant further evaluation by larger multicenter RCTs to determine the efficacy and safety profiles of DEB-TACE method in comparison to the C-TACE treatment.

## Figures and Tables

**Table 1:** Study characteristics of included studies.

Study Year	Country	Study Design	Gender (Male/Female)	Number of Procedures	DEB-TACE	C-TACE	Age † (Years)	Length of Stay (Months)
Dhanasekaran et al., 2010	USA	Case–control	54/17	71	45	26	59.6 ± 12.1	3–6
Kloeckner et al., 2015	Germany	Case–control	212/38	250	76	174	NR	18
Wiggermannet al., 2011	Germany	Case–control	37/7	44	22	22	70.3 ± 7.1	1.5–2
Nicolini et al., 2013	Italy	Case–control	34/4	38	22	16	56.5 ± 6.5	NR
Scartozzi et al., 2010	Italy	Case–control	122/28	150	87	63	69 (40–89)	NR
Arabi et al., 2015	KSA	Case–control	39/16	76	51	25	67	3
Song et al., 2012	South Korea	Case–control	90/39	129	60	69	60.5 ± 10.6	18
Kucukay et al., 2015	Turkey	Case–control	103/23	126	53	73	63.8 ± 10.9	12
Frenette et al., 2014	USA	Case–control	91/20	111	35	76	59.2 ± 7.9	NR
Hui Liet al., 2019	China	Cohort	70/11	81	42	39	57.1 ± 14.1	15
Ganget al., 2017	China	Cohort	36/6	42	22	20	NR	6
Wenet al., 2019	China	Cohort	99/21	120	52	68	58.9 ± 12.1	NR
Facciorusso et al., 2015	Italy	Cohort	197/52	249	145	104	67 (67–93)	42
Recchia et al., 2012	Italy	Cohort	75/30	105	35	70	71 (47–80)	14
Morimoto et al., 2016	Japan	Cohort	78/27	105	50	55	72.4 ± 9.7	19
Petruzzi et al., 2013	USA	Cohort	51/12	63	33	30	64 (25–82)	9.6
Soo Lee et al., 2016	South Korea	Cohort	89/19	108	54	54	63.3 ± 10.4	36.8
Lee et al., 2016	South Korea	Cohort	204/46	250	144	106	62 (30–90)	NR
Puchol et al., 2011	Spain	Cohort	NR	72	47	25	69.3 ± 11.8	1.
Monier et al., 2016	Switzerland	Cohort	113/18	131	74	57	64.2 ± 11.8	27 ± 23
Elshahat et al., 2015	Egypt	Cohort	40/20	60	34	26	61.1 (32–81)	6
Massani et al., 2017	Italy	Cohort	69/13	82	28	54	68.3 ± 11.3	12
Rahman et al., 2016	Malaysia	Cohort	62/17	79	45	34	62 ± 11	11.8
Van et al., 2011	Europe	RCT	25/5	30	16	14	62.3 ± 12.6	1.5
Vogl et al., 2011	Germany	RCT	185/27	212	102	110	67.0 ± 9.2	6
Lammer et al., 2010	Germany	RCT	174/27	201	93	108	67.3 ± 9.1	6
Golfieri et al., 2014	Italy	RCT	135/42	177	89	88	68.6 ± 8.0	24
Sacco et al., 2011	Italy	RCT	45/22	67	33	34	70 ± 7.7	26.8 ± 12
Bargellini et al., 2021	Italy	Cohort	163/39	202	101	101	62.7 ± 10.8	2.5 ± 2.3
Fan et al., 2021	China	Cohort	107/12	119	57	62	50 ± 11	6 (4–8) *
Zhang et al., 2021	China	Cohort	871/131	1002	394	608	60 ± 13	NR
Chiu et al., 2020	Taiwan	Cohort	50/11	61	42	19	65 (27–87.6)	NR
Shimose et al., 2020	Japan	Cohort	111/63	174	76	98	73 (51–91)	NR
Wu et al., 2018	China	Cohort	49/5	54	24	30	55.2 ± 8.5	NR

† Age reported as mean ± SD where indicated. * Length of stay reported as days where indicated. Abbreviations: C-TACE: conventional transcatheter arterial chemoembolization; DEB-TACE: drug-eluting beads transcatheter arterial chemoembolization; KSA: Kingdom of Saudi Arabia; NR: not reported; RCT: randomized controlled trial; USA: United States of America.

**Table 2:** Summary of the pooled analyses for the efficacy and safety outcomes.

Outcome	No. of Studies	Test for Heterogeneity			Test of Association		
		Tau2	*p*-Value	I2 (%)	OR (95% CI)	z	*p*-Value
**Complete Response**	23	0.3515	<0.01	67	1.27 (0.91, 1.76)	1.42	0.15
Partial response	21	0.1409	0.01	46	1.08 (0.91, 1.28)	0.93	0.35
Objective response rate	20	0.2563	<0.01	60	1.33 (0.99, 1.79)	1.89	0.06
Disease stability	19	0.4499	<0.01	60	0.82 (0.55, 1.22)	−0.97	0.33
Disease control	16	0.1883	<0.01	51	1.42 (1.03, 1.96)	2.16	**<0.05**
Disease progression	20	0.5381	<0.01	63	0.80 (0.52, 1.22)	−1.05	0.29
Systemic adverse events	5	1.2525	<0.01	83	0.74 (0.24, 2.24)	−0.54	0.59
Serious adverse events	18	0.1505	0.11	31	0.96 (0.79, 1.17)	−0.41	0.68
30-day mortality	7	0	0.99	0	0.48 (0.21, 1.10)	−1.73	0.08
End mortality	7	0.4344	0.04	54	0.32 (0.16, 0.65)	−3.15	**<0.01**

**Bold** font indicates significance set at *p* < 0.05 or < 0.01.

## Supplementary Materials

The following are available online at https://www.mdpi.com/article/10.3390/cancers13246172/s1, Table S1: Comparison between the criteria for tumor response assessments. Table S2: Search strategy.

## References

[1] Fitzmaurice C., Allen C., Barber R.M., Barregard L., Bhutta Z.A., Brenner H., Dicker D.J., Chimed-Orchir O., Dandona R., Dandona L. (2017). Global, Regional, and National Cancer Incidence, Mortality, Years of Life Lost, Years Lived with Disability, and Disability-Adjusted Life-years for 32 Cancer Groups, 1990 to 2015: A Systematic Analysis for the Global Burden of Disease Study. JAMA Oncol..

[2] Rawla P., Sunkara T., Muralidharan P., Raj J.P. (2018). Update in global trends and aetiology of hepatocellular carcinoma. Contemp. Oncol..

[3] El-Serag H.B., Mason A.C. (1999). Rising Incidence of Hepatocellular Carcinoma in the United States. N. Engl. J. Med..

[4] Balogh J., Victor D., Asham E.H., Burroughs S.G., Boktour M., Saharia A., Li X., Ghobrial R.M., Monsour H.P. (2016). Hepatocellular carcinoma: A review. J. Hepatocell. Carcinoma.

[5] Mohammadian M., Mahdavifar N., Mohammadian-Hafshejani A., Salehiniya H. (2018). Liver cancer in the world: Epidemiology, incidence, mortality and risk factors. World Cancer Res. J..

[6] Golabi P., Fazel S., Otgonsuren M., Sayiner M., Locklear C.T., Younossi Z.M. (2017). Mortality assessment of patients with hepatocellular carcinoma according to underlying disease and treatment modalities. Medicine.

[7] Forner A., Reig M.E., de Lope C.R., Bruix J. (2010). Current strategy for staging and treatment: The BCLC update and future prospects. Semin. Liver Dis..

[8] Nouri Y.M., Kim J.H., Yoon H.-K., Ko H.-K., Shin J.H., Gwon D.I. (2019). Update on Transarterial Chemoembolization with Drug-Eluting Microspheres for Hepatocellular Carcinoma. Korean J. Radiol..

[9] Miyayama S. (2020). Treatment Strategy of Transarterial Chemoembolization for Hepatocellular Carcinoma. Appl. Sci..

[10] Kalva S.P., Iqbal S.I., Yeddula K., Blaszkowsky L.S., Akbar A., Wicky S., Zhu A.X. (2011). Transarterial chemoembolization with Doxorubicin-eluting microspheres for inoperable hepatocellular carcinoma. Gastrointest. Cancer Res..

[11] De Baere T., Arai Y., Lencioni R., Geschwind J.-F., Rilling W., Salem R., Matsui O., Soulen M.C. (2016). Treatment of liver tumors with lipiodol TACE: Technical recommendations from experts opinion. Cardiovasc. Intervent. Radiol..

[12] Varela M., Real M.I., Burrel M., Forner A., Sala M., Brunet M., Ayuso C., Castells L., Montañá X., Llovet J.M. (2007). Chemoembolization of hepatocellular carcinoma with drug eluting beads: Efficacy and doxorubicin pharmacokinetics. J. Hepatol..

[13] Li H., Wu F., Duan M., Zhang G. (2019). Drug-eluting bead transarterial chemoembolization (TACE) vs. conventional TACE in treating hepatocellular carcinoma patients with multiple conventional TACE treatments history. Medicine.

[14] Golfieri R., Giampalma E., Renzulli M., Cioni R., Bargellini I., Bartolozzi C., Breatta A.D., Gandini G., Nani R., Gasparini D. (2014). Randomised controlled trial of doxorubicin-eluting beads vs. conventional chemoembolisation for hepatocellular carcinoma. Br. J. Cancer.

[15] Recchia F., Passalacqua G., Filauri P., Doddi M., Boscarato P., Candeloro G., Necozione S., Desideri G., Rea S. (2012). Chemoembolization of unresectable hepatocellular carcinoma: Decreased toxicity with slow-release doxorubicineluting beads compared with lipiodol. Oncol. Rep..

[16] Sacco R., Bargellini I., Bertini M., Bozzi E., Romano A., Petruzzi P., Tumino E., Ginanni B., Federici G., Cioni R. (2011). Conventional versus doxorubicin-eluting bead transarterial chemoembolization for hepatocellular carcinoma. J. Vasc. Intern. Radiol..

[17] Van Malenstein H., Maleux G., Vandecaveye V., Heye S., Laleman W., van Pelt J., Vaninbroukx J., Nevens F., Verslype C. (2011). A randomized phase II study of drug-eluting beads versus transarterial chemoembolization for unresectable hepatocellular carcinoma. Onkologie.

[18] Scartozzi M., Baroni G.S., Faloppi L., Paolo M.D., Pierantoni C., Candelari R., Berardi R., Antognoli S., Mincarelli C., Risaliti A. (2010). Trans-arterial chemo-embolization (TACE), with either lipiodol (traditional TACE) or drug-eluting microspheres (precision TACE, pTACE) in the treatment of hepatocellular carcinoma: Efficacy and safety results from a large mono-institutional analysis. J. Exp. Clin. Cancer Res..

[19] Song M.J., Chun H.J., Song D.S., Kim H.Y., Yoo S.H., Park C.H., Bae S.H., Choi J.Y., Chang U.I., Yang J.M. (2012). Comparative study between doxorubicin-eluting beads and conventional transarterial chemoembolization for treatment of hepatocellular carcinoma. J. Hepatol..

[20] Dhanasekaran R., Kooby D.A., Staley C.A., Kauh J.S., Khanna V., Kim H.S. (2010). Comparison of conventional transarterial chemoembolization (TACE) and chemoembolization with doxorubicin drug eluting beads (DEB) for unresectable hepatocelluar carcinoma (HCC). J. Surg. Oncol..

[21] Lammer J., Malagari K., Vogl T., Pilleul F., Denys A., Watkinson A., Pitton M., Sergent G., Pfammatter T., Terraz S. (2010). Prospective randomized study of doxorubicin-eluting-bead embolization in the treatment of hepatocellular carcinoma: Results of the PRECISION V study. Cardiovasc. Intervent. Radiol..

[22] Meyer T., Kirkwood A., Roughton M., Beare S., Tsochatzis E., Yu D., Davies N., Williams E., Pereira S.P., Hochhauser D. (2013). A randomised phase II/III trial of 3-weekly cisplatin-based sequential transarterial chemoembolisation vs. embolisation alone for hepatocellular carcinoma. Br. J. Cancer.

[23] Malagari K., Pomoni M., Kelekis A., Pomoni A., Dourakis S., Spyridopoulos T., Moschouris H., Emmanouil E., Rizos S., Kelekis D. (2010). Prospective randomized comparison of chemoembolization with doxorubicin-eluting beads and bland embolization with BeadBlock for hepatocellular carcinoma. Cardiovasc. Intervent. Radiol..

[24] Chen P., Yuan P., Chen B., Sun J., Shen H., Qian Y. (2017). Evaluation of drug-eluting beads versus conventional transcatheter arterial chemoembolization in patients with unresectable hepatocellular carcinoma: A systematic review and meta-analysis. Clin. Res. Hepatol. Gastroenterol..

[25] Facciorusso A., Di Maso M., Muscatiello N. (2016). Drug-eluting beads versus conventional chemoembolization for the treatment of unresectable hepatocellular carcinoma: A meta-analysis. Dig. Liver Dis..

[26] Zou J.H., Zhang L., Ren Z.G., Ye S.L. (2016). Efficacy and safety of cTACE versus DEB-TACE in patients with hepatocellular carcinoma: A meta-analysis. J. Dig. Dis..

[27] Xie Z.B., Wang X.B., Peng Y.C., Zhu S.L., Ma L., Xiang B.D., Gong W.-F., Chen J., You X.-M., Jiang J.-H. (2015). Systematic review comparing the safety and efficacy of conventional and drug-eluting bead transarterial chemoembolization for inoperable hepatocellular carcinoma. Hepatol. Res..

[28] Huang K., Zhou Q., Wang R., Cheng D., Ma Y. (2014). Doxorubicin-eluting beads versus conventional transarterial chemoembolization for the treatment of hepatocellular carcinoma. J. Gastroenterol. Hepatol..

[29] Gao S., Yang Z., Zheng Z., Yao J., Deng M., Xie H., Zheng S., Zhou L. (2013). Doxorubicin-eluting bead versus conventional TACE for unresectable hepatocellular carcinoma: A meta-analysis. Hepatogastroenterology.

[30] Wang H., Cao C., Wei X., Shen K., Shu Y., Wan X., Sun J., Ren X., Dong Y., Liu Y. (2020). A comparison between drug-eluting bead-transarterial chemoembolization and conventional transarterial chemoembolization in patients with hepatocellular carcinoma: A meta-analysis of six randomized controlled trials. J. Can. Res. Ther..

[31] Liang B., Makamure J., Shu S., Zhang L., Sun T., Zheng C. (2021). Treatment Response, Survival, and Safety of Transarterial Chemoembolization with CalliSpheres^®^ Microspheres versus Conventional Transarterial Chemoembolization in Hepatocellular Carcinoma: A Meta-Analysis. Front. Oncol..

[32] Moher D. (2009). Preferred Reporting Items for Systematic Reviews and Meta-Analyses: The PRISMA Statement. Ann. Internal Med..

[33] DerSimonian R., Laird N. (1986). Meta-analysis in clinical trials. Control. Clin. Trials.

[34] Begg C.B., Mazumdar M. (1994). Operating characteristics of a rank correlation test for publication bias. Biometrics.

[35] Burrel M., Reig M., Forner A., Barrufet M., de Lope C.R., Tremosini S., Ayuso C., Llovet J.M., Real M.I., Bruix J. (2012). Survival of patients with hepatocellular carcinoma treated by transarterial chemoembolisation (TACE) using Drug Eluting Beads. Implications for clinical practice and trial design. J. Hepatol..

[36] Oliveri R.S., Wetterslev J., Gluud C. (2011). Transarterial (chemo) embolisation for unresectable hepatocellular carcinoma. Cochrane Database Syst. Rev..

[37] Boatta E., Corona M., Cannavale A., Fanelli F., Cirelli C., de Medici L. (2013). Endovascular treatment of hepatocellular carcinoma with drug eluting microparticles (DC-Beads): CT evaluation of response to the treatment. Indian J. Radiol. Imaging.

