# Supplementary material for: Conventional Transarterial Chemoembolization Versus Drug-Eluting Beads in Patients with Hepatocellular Carcinoma: A Systematic Review and Meta-Analysis
Source: Cancers (Basel). 2021 Dec 7;13(24):6172. doi: 10.3390/cancers13246172 (PMC8699068; doi:10.3390/cancers13246172)
Supplement: Supplementary file 1 [file cancers-13-06172-s001.zip › cancers-1426867-supplementary.pdf]

Table S1. Comparison between the criteria for tumor response assessments

Tumor Response	mRECIST	EASL criteria
**Complete response**	Disappearance of all target lesions	Absence of enhanced tumor areas, reflecting complete tumor necrosis
**Partial response**	≥30% decrease	≥50% decrease of enhanced areas
**Objective response**	Complete and partial response	Complete and partial response
**Disease control**	Complete, partial, and stable disease response	Complete, partial, and stable disease response
**Stable disease**	≤30% decrease and and≤20% increase	≤50% decrease and ≤25% increase of enhanced areas
**Progressive disease**	≥20% increase or new lesions	≥25% increase of enhanced lesions or new enhanced lesions

EASL = European Association for the Study of the Liver, mRECIST = modified Response Evaluation Criteria in Solid Tumors

Table S2. Search Strategy

No.	Query	Description
**#1**	"liver neoplasms"[MeSH] OR ("biliary tract" OR "hepatobiliary" OR "liver") AND (cancer* OR carcinom* OR neoplasm* OR tumor* OR sarcoma* OR tumour*) OR "ascites hepatomas" OR "hepatoblastomas" OR "liver carcinogenesis" OR "morris hepatomas" OR "novikoff hepatomas"	**Search terms:** Hepatocellular Carcinoma
**#2**	"DEB" OR "drug-eluting microsphere" OR "drug-eluting" OR drug-elut*	**Search terms:** Drug-Eluting
**#3**	("TACE" OR "transcatheter arterial chemoembolization" OR "transarterial chemoembolization") OR (therapeutic chemoembolization[MeSH Terms])	**Search terms:** Conventional
